# Nonsteroidal Anti-Inflammatory Drugs for Retinal Disease

**DOI:** 10.1155/2013/281981

**Published:** 2013-01-14

**Authors:** Scott D. Schoenberger, Stephen J. Kim

**Affiliations:** Vanderbilt Eye Institute, Vanderbilt University Medical Center, Nashville, TN 37232, USA

## Abstract

Nonsteroidal anti-inflammatory drugs (NSAIDs) are used extensively in ophthalmology for pain and photophobia after photorefractive surgery and to reduce miosis, inflammation, and cystoid macular edema following cataract surgery. In recent years, the US Food and Drug Administration has approved new topical NSAIDs and previously approved NSAIDs have been reformulated. These changes may allow for greater drug penetration into the retina and thereby offer additional therapeutic advantages. For example, therapeutic effects on diabetic retinopathy and age-related macular degeneration may now be achievable. We provide an updated review on the scientific rationale and clinical use of NSAIDs for retinal disease.

## 1. Introduction

Nonsteroidal anti-inflammatory drugs (NSAIDs) are one of the most commonly prescribed classes of medications and are routinely employed for their analgesic, antipyretic, and anti-inflammatory properties. NSAIDs are potent inhibitors of cyclooxygenase (COX) enzymes and thereby the synthesis of pro-inflammatory prostaglandins (PGs). In ophthalmology, topical NSAIDs are used to stabilize pupillary dilation during intraocular surgery and to treat allergic conjunctivitis and postoperative inflammmation, pain and cystoid macular edema (CME) [1]. The therapeutic efficacy of topical NSAIDs for these aforementioned conditions has been well established [1, 2]. There is also increasing evidence that PGs play a role in the pathogenesis of diabetic retinopathy and age-related macular degeneration (AMD) and recent years have seen more studies examining the therapeutic role of NSAIDs for these disorders [1]. The intent of this paper is to focus on the potential application of NSAIDs to treat retinal disease.

## 2. Nonsteroidal Anti-Inflammatory Drugs

NSAIDs are a class of medications that lack a steroid nucleus and inhibit COX enzymes [1]. COX enzymes catalyze the production of five classes of PGs: PGE_2_, PGD_2_, PGF_2*α*_, PGI_2_, and Thromboxane A_2_. Two main isoforms of COX, COX-1 and COX-2, exist [3], and a third (COX-3) remains largely uncharacterized [4]. COX-1 contributes to normal physiological processes and is expressed in the gastrointestinal tract, kidneys, platelets, and vascular endothelium [1]. COX-2 is an inducible enzyme that is upregulated during pain, fever, and inflammatory responses, but is also expressed in some systems under normal conditions. COX-2 is the predominate isoform in retinal pigment epithelium (RPE) cells and is up-regulated in the presence of proinflammatory cytokines [5]. COX-2 has an important role in angiogenesis and has been implicated in choroidal neovascularization (CNV) and proliferative diabetic retinopathy (PDR) [1].

PGs are an important class of inflammatory mediators that are biosynthesized from membrane bound arachidonic acid. Within the eye, PGs disrupt the blood-ocular barrier, increase vasodilation, and facilitate leukocyte migration [1]. They also interact with and amplify many other soluble mediators including vascular endothelial growth factor (VEGF) [1, 6, 7]. As a result, their inhibition has favorable effects on intraocular inflammation and retinal edema [8]. 

### 2.1. Formulations

Several topical NSAIDs are commercially available for ophthalmic use, including ketorolac, diclofenac, nepafenac, bromfenac, and flurbiprofen. Dosing varies from daily (Bromday, bromfenac 0.09%, ISTA Pharmaceuticals) to four times daily (Acular, ketorolac 0.5%, Allergan, Inc.). Ketorolac is reported to be the most potent inhibitor of COX-1, while bromfenac and amfenac are the most potent inhibitors of COX-2 [9–13]. Bromfenac may be 3 to 18 fold more potent of an inhibitor of COX-2 than diclofenac, ketorolac and amfenac (the active metabolite of nepafenac) [9, 12], but this attribute has not been consistently reported [13]. Furthermore, the relative importance of COX-1 versus COX-2 inhibition in ocular disease remains unproven [1].

### 2.2. Aqueous Levels

Several studies have measured intraocular NSAID levels in humans after topical use. After a single application, peak aqueous drug levels are detectable for: diclofenac 0.1% (82 ng/mL; 2.4 hour peak), flurbiprofen 0.03% (60 ng/mL; 2.0 hour peak), nepafenac 0.1% (205.3 ng/mL; peak 30 minutes), amfenac (70.1 ng/mL), ketorolac 0.4% (57.5 ng/mL; 60 minutes), and bromfenac 0.09% (25.9 ng/mL) [13, 14]. Acuvail (Allergan, Inc) is a newer preservative-free formulation (0.45%) of ketorolac dosed twice daily that has been reported to achieve a much higher peak aqueous concentration after a single application than older formulations but as of yet has not been tested in humans [15]. More frequent and continued dosing leads to even higher aqueous levels. Twelve doses over two days of ketorolac 0.4% and nepafenac 0.1% result in reported aqueous levels of 1079 ng/mL of ketorolac and 353.4 ng/mL of amfenac [16], which far exceed reported inhibitory concentration 50 (IC_50_) for COX-1 and COX-2 enzymes for both NSAIDs: ketorolac (COX-1, 5.3 to 7.5 ng/mL; COX-2, 33.9 to 45.2 ng/mL) and amfenac (COX-1, 35.6 to 63.6 ng/mL; COX-2, 0.51 to 38.1 ng/mL).

### 2.3. Vitreous Levels

In contrast to aqueous drug levels, there is a paucity of human studies measuring NSAID levels in the vitreous after topical application. A single study measured vitreous drug levels in patients who received ketorolac 0.4% four times daily, bromfenac 0.09% two times daily, or nepafenac 0.1% three times daily for three days before vitrectomy surgery [17]. Vitreous levels of ketorolac, bromfenac, and amfenac were reported as 2.8 ng/mL, 0.96 ng/mL, and 2.0 ng/mL, respectively, but only ketorolac resulted in significantly lower vitreous PGE_2_ levels compared to placebo. Aqueous and vitreous concentrations of NSAID would likely have a direct effect on anterior (ciliary body and iris) and posterior (retina and choroid) PG production, respectively.

## 3. Postoperative Cystoid Macular Edema

Cystoid macular edema is the accumulation of extracellular fluid within the retina due to leakage from dilated capillaries. It is the most common cause of vision loss after cataract surgery [1], and was first described over a half-century ago [18]. Its incidence has been reported to be as high as 9–19% on fluorescein angiography (FA) and 41% on optical coherence tomography (OCT), but clinically important CME is far less common [19–21]. Inflammation has been implicated as a main cause of postoperative CME [1] and numerous studies have examined the role of NSAIDs for the treatment of acute and chronic CME and its prophylaxis.

### 3.1. Acute and Chronic CME

CME associated with cataract surgery may be treated early (less than 6 months) or late (6 months or more) following its diagnosis [1]. These two groups are distinguished as acute and chronic CME. The efficacy of topical NSAIDs in treating both conditions has been reviewed in great detail elsewhere with general consensus, despite the paucity of well-designed studies, that treatment with NSAIDs is beneficial (reduces macular edema and may improve vision) at least over the short-term [1]. Recently, Warren et al. evaluated the adjunctive use of nepafenac 0.1%, diclofenac 0.1%, ketorolac 0.4%, bromfenac 0.09%, or placebo in 39 patients for 16 weeks in addition to intravitreal triamcinolone and bevacizumab for treatment of chronic CME [22]. Both adjunctive use of nepafenac and bromfenac resulted in greater reduction of retinal thickness at 12 and 16 weeks but only nepafenac led to a significant improvement in vision. Similarly, in a retrospective, uncontrolled study, nepafenac 0.1% improved retinal thickness and visual acuity in patients with chronic, recalcitrant CME [23].

### 3.2. Prophylaxis of CME

Numerous studies have evaluated NSAIDs for prevention of postoperative CME following cataract surgery. Only pertinent well-designed studies are reviewed here. A randomized, double-masked, placebo-controlled trial by Flach et al. reported that prophylactic use of ketorolac 0.5% was effective in reducing angiographic CME in aphakic patients without the use of corticosteroids [24]. A multicenter, prospective study compared the effects of topical diclofenac 0.1% versus fluorometholone (FML) 0.1% on prevention of CME in eyes undergoing modern, small-incision phacoemulsification [25]. Five weeks after surgery, angiographic CME was present in 5.7% of diclofenac-treated eyes and 54.7% of FML-treated eyes. FML has limited intraocular penetration; therefore, these results may approximate the effectiveness of diclofenac as compared to placebo. A more recent randomized, masked comparison of topical ketorolac 0.4% plus corticosteroid versus corticosteroid alone demonstrated a significantly reduced rate of CME with combination treatment in low-risk patients after cataract surgery [26]. However, the absolute incidence of definite or probable CME was low in both groups (2.4% for corticosteroid group; 0% for ketorolac/corticosteroid group) and there was no difference reported in visual outcomes. The results of this latter study question the cost-effectiveness of routine prophylactic treatment with both a corticosteroid and NSAID for patients at low risk for CME. On the other hand, routine use in patients with diabetes or uveitis who are at higher risk of developing postoperative CME may be warranted [27]. 

The use of a topical NSAID and corticosteroid together is sometimes reported to be “synergistic” in the literature. This clinical impression of synergy remains unproven and would seem unlikely given the fact that both drug classes have overlapping mechanisms of action [8]. Synergy is defined as two or more agents working in combination to produce an effect that could not be obtained by either agent alone. A classic example of synergy involves penicillin and aminoglycoside antibiotics where use of both antibiotics in combination significantly lowers the IC_50_ of each antibiotic for a given microorganism. Although a large, randomized, prospective study demonstrated that ketorolac 0.5% was more effective than dexamethasone sodium phosphate 0.1% solution in facilitating reestablishment of the blood-aqueous barrier after surgery, differences in drug formulation and intraocular concentration preclude any conclusions about synergy [28]. Furthermore, although many prospective studies have confirmed that the combination use of a NSAID and corticosteroid is superior to a corticosteroid alone for CME and visual improvement after intraocular surgery, these findings can be explained by an additive effect of a second anti-inflammatory agent.

### 3.3. CME after Vitreoretinal Surgery

Several studies have assessed the therapeutic benefit of NSAIDs for the prevention of CME after vitreoretinal surgery. A prospective, randomized, placebo-controlled trial reported that topical ketorolac 0.4% reduced both retinal thickness (9%) and total macular volume (6%) but neither outcome reached statistical significance [29]. Schoenberger et al. reported that topical nepafenac more rapidly reduced macular volume in patients undergoing epiretinal membrane surgery, but this effect was not observed by another study using nepafenac [30, 31].

## 4. Age-Related Macular Degeneration

CNV is the most common cause of severe vision loss in patients with the wet (neovascular) form of age-related macular degeneration (AMD) [32–34]. AMD is the leading cause of blindness in the United States and will affect nearly 8 million Americans by 2020 [32]. Many patients with AMD have moderate vision loss (20/50 to 20/100) in the better eye that results in quality-of-life measurements that are 32% below normal and similar to patients with severe angina or hip fractures [33]. An increasing percentage of patients with AMD suffer severe vision loss (20/800) which results in a 60% reduction in quality of life and is similar to a patient who is bedridden due to a catastrophic stroke.

It is now firmly established that VEGF is a principle mediator of CNV. While VEGF inhibitors have been an important advance in treating neovascular AMD, they do not slow down the underlying disease process. Moreover, VEGF is essential for normal homeostasis of retinal cells and its chronic inhibition may therefore be undesirable [35]. Consequently, it is clear that strictly inhibiting VEGF neither addresses the multifactorial pathogenesis of CNV nor the underlying cause of VEGF induction. Instead, a growing body of scientific evidence indicates that inflammation plays a central role in CNV [36, 37]. A better understanding of inflammatory mediators of VEGF induction may therefore provide an opportunity to develop preventative strategies.

In this regard, COX-2 can be detected in human choroidal neovascular membranes [38] and considerable scientific evidence indicates that COX is a promoter of angiogenesis [39, 40]. Patients who regularly take NSAIDs have a 40–50% reduction in mortality from colorectal cancer and a distinguishing feature of colorectal tumors is high expression of COX [41]. Pharmacologic inhibition of COX appears to reduce VEGF expression in cultured human RPE cells and suppresses VEGF in both trauma- and ischemia-induced models of retinal angiogenesis [42–44]. In a variety of experimental systems, inhibition of COX-2 suppresses angiogenesis. *In vitro* studies have demonstrated that PGE_2_ increases VEGF expression in cultured Müller cells and agonism or antagonism of the PGE_2_ receptor EP_4_ increases or decreases VEGF production, respectively [42].

### 4.1. Animal Studies

Animal studies have consistently shown that NSAIDs reduce or inhibit CNV. Kim et al. have demonstrated that both topical and intravitreal ketorolac significantly reduces angiographic leakage and retinal levels of PGE_2_ and VEGF in an animal model of CNV [45, 46]. Furthermore, CNV was significantly reduced in COX-2 null mice after laser-induction, an effect that could be explained by reduced retinal VEGF [47]. Other investigators have also independently reported similar observations with administration of topical or oral NSAIDs [48, 49].

### 4.2. Clinical Studies

In contrast to more robust evidence in animal studies, clinical evidence demonstrating a consistent therapeutic benefit of NSAIDs for AMD is lacking. A cohort of patients with rheumatoid arthritis was prospectively followed and found to have a low prevalence of AMD [50], presumed to be due to long-term administration of anti-inflammatory medications, and a large retrospective study reported decreased rates of CNV among AMD patients taking aspirin [51]. In contrast, no association between systemic NSAIDs and five-year incidence of age-related maculopathy was observed in the Blue Mountains Eye Study [52].

Studies investigating topical NSAIDs for exudative AMD (Table 1) [53–58] have also reported conflicting results. A randomized, controlled study reported no additional benefit in regards to vision or lesion size with combination treatment with diclofenac and photodynamic therapy for subfoveal CNV [55]. Two retrospective studies also showed no benefit with the addition of topical bromfenac or nepafenac to intravitreal anti-VEGF agents in patients with persistently active exudative AMD [53, 54]. In contrast, two prospective, randomized, controlled clinical studies reported favorable effects of topical bromfenac with respect to retinal thickness and reduced number of anti-VEGF treatments. Flaxel et al. investigated combination treatment with topical bromfenac 0.09% for new or recurrent exudative AMD [57]. Patients received monthly intravitreal ranibizumab (IVR) for four months, followed by as needed treatment and were randomized to either combination treatment with bromfenac or monotherapy. There was no observed difference in regards to vision or number of injections between groups, but there was a significant difference in favor of combination treatment in reduction of central macular thickness (−81.56 microns, combination group; −42.50 microns, IVR group). In an independent study by Gomi et al., combination treatment with bromfenac 0.1% and IVR significantly reduced the number of anti-VEGF injections needed compared to IVR monotherapy [58].

## 5. Diabetic Macular Edema and Diabetic Retinopathy

Diabetic retinopathy (DR) is the most frequent cause of legal blindness among working-aged individuals in developed countries [59]. Diabetic macular edema (DME) is the most common cause of vision loss in diabetic patients, affecting about 75,000 new patients in the United States every year [60]. Proven preventable measures for DR include lowering of high blood pressure and strict control of blood glucose [61, 62] but a growing body of scientific evidence supports a pathogenic role of inflammation [63].

In support of this, a number of pro-inflammatory cytokines are consistently elevated in the vitreous of patients with advanced stages of DR [64–66] and treatment with NSAIDs prevents or delays its progression in animal models. Recent work from our group has demonstrated elevated levels of PGE_2_ in vitreous samples taken from patients with PDR which correlate with vitreous levels of VEGF and provides support for a pathogenic role of PGs in DR [67].

### 5.1. Experimental and Animal Studies

In both experimental and animal models, PGs induce VEGF production [45, 68] with subsequent development of vascular leakage and retinal neovascularization [69]. In cultured Müller cells, agonism or antagonism of the PGE_2_ receptor EP_4_ increases or decreases VEGF production, respectively, in a dose-dependent manner [42]. Retinal cells consistently upregulate COX and PGs [43, 70] in DR and PGE_2_ is increased by 40% in the retinal vasculature of diabetic rats [70]. Topical nepafenac 0.1% significantly inhibits diabetes-induced retinal microvascular disease and treatment with celecoxib reduces retinal VEGF expression and vascular leakage in streptozotocin-induced diabetic rats [71, 72]. Administration of other NSAIDs (nepafenac, aspirin, meloxicam) has also been reported to inhibit diabetes-induced retinal microvascular disease and prevent early DR [71, 73].

### 5.2. Systemic Therapy

The therapeutic benefit of systemic NSAIDs for DR has been evaluated in a few clinical studies. It was first observed a half century ago that rheumatoid arthritis patients taking salicylates had a reduced incidence of DR [74]. This observation was later examined in two large multicenter clinical trials, the Early Treatment Diabetic Retinopathy Study (ETDRS), which examined the effect of 650 mg aspirin on advanced DR [75], and the Dipyridamole Aspirin Microangiopathy of Diabetes (DAMAD) Study [76], which tested the impact of 990 mg aspirin in patients with early DR. While no benefit was found in patients with more advanced DR in ETDRS, a significant effect was seen in the DAMAD study, where higher doses of aspirin were found to slow the development of retinal microaneurysms. This latter observation is supported by a randomized 3-year pilot study where the NSAID sulindac prevented development and progression of DR [77]. Similarly, a recent prospective, controlled trial conducted by the National Eye Institute demonstrated that oral celecoxib significantly reduced vascular leakage in patients with DR despite premature stoppage of treatment due to concerns regarding cardiovascular toxicity [78]. Finally, a recent randomized clinical trial by the Diabetic Retinopathy Clinical Research (DRCR) Network reported that intravitreal injection of corticosteroid (triamcinolone acetonide) significantly reduced progression of DR, which provides further support for anti-inflammatory based therapies [79].

### 5.3. Topical Therapy

There are uncontrolled case reports reporting anatomical and visual improvement with topical NSAIDs for DME. Hariprasad et al. described several patients with macular edema (most had CME) that were treated with nepafenac 0.1% [80]. One patient underwent treatment for DME for six months with improved retinal thickness from 378 microns to 215 microns and a three-line improvement in visual acuity (VA). In another study, six eyes of five patients were treated with nepafenac 0.1% for DME for a mean duration of 210 days [81]. Median logarithm of the minimal angle of resolution (logMAR) VA statistically improved from 0.78 at baseline to 0.67 at the final visit. Mean foveal thickness statistically improved from 417 microns at baseline to 267 microns. A phase II, randomized, double-blinded study is currently recruiting participants to receive placebo or nepafenac 0.1% for 12 months in the treatment of noncentral DME [82]. 

### 5.4. Intravitreal Therapy

Four studies have evaluated intravitreal diclofenac or ketorolac for DME (Table 2). Soheilian et al. investigated the safety and efficacy of a single intravitreal injection of diclofenac (500 mcg/0.1 mL) in five eyes with DME [83]. After eight weeks, VA improved in two eyes, worsened in two eyes, and remained stable in one eye, while mean central macular thickness (CMT) was actually worse than at baseline. Elbendary and Shahin compared intravitreal diclofenac (500 mcg/0.1 mL) to intravitreal triamcinolone (4 mg/0.1 mL) in the treatment of diffuse DME in a randomized study [84]. CMT decreased in the diclofenac group from 419.8 microns at baseline to 323.5 microns at one month and 271.1 microns at three months. There was no difference between the two groups in CMT, final VA, mean line improvement, and percent of eyes with improved VA. Reis Ado et al. treated twenty patients with bilateral DME refractory to laser therapy [85]. One eye received intravitreal ketorolac (500 mcg/0.1 mL), while the other served as a control. At one month, there was a significant improvement in VA in the treated eyes relative to controls, but there was no change in foveal thickness or macular volume. Maldonado et al. treated 25 patients with DME refractory to laser with a single injection of ketorolac (3000 mcg/0.1 mL). At one month, 28% of patients had an improvement in VA of at least five letters, while there was no significant difference in macular thickness [86].

## 6. Conclusions

Although there is good collective evidence that topical NSAIDs treat and prevent CME after cataract surgery, the long-term visual benefits of this practice remain unknown since CME can resolve spontaneously. It is now well established that inflammation plays a pathogenic role in AMD, DR, and DME, but clinical data demonstrating a therapeutic effect of NSAIDs for these diseases is limited and derived mostly from small, retrospective or uncontrolled studies. Despite considerable scientific rationale, there is insufficient evidence to recommend using NSAIDs to treat these conditions until more compelling clinical data emerges.

## Figures and Tables

**Table 1:** Studies that investigated topical NSAIDs for exudative AMD.

Study	Design, sample size and study duration	NSAID	Treatment group(s)	Outcomes	Author conclusions
Boyer et al. (2007) [55]	Randomized, prospective, placebo-controlled 57 eyes 3 months	Diclofenac 0.1%	Diclofenac with PDT (C) versus PDT for subfoveal classic CNV	No improvement in VA, lesion area, GLD, fluorescein leakage, or CMT	No added benefit of diclofenac to PDT for subfoveal classic CNV

Grant (2008) [56]	Retrospective, comparative 60 eyes 6 months	Bromfenac 0.09%	Bromfenac with IVR (C) versus IVR for wet AMD	VA increased more in C group (*P* = 0.001) Fewer injections in C group (*P* = 0.0002)	Combination therapy with bromfenac may be more efficacious than IVR alone

Zweifel et al. (2009) [53]	Retrospective, uncontrolled 22 eyes 2 months	Bromfenac 0.09%	Bromfenac with IVR/IVB for persistent SRF/IRF	VA and CMT unchanged at end of study	No added benefit of bromfenac to standard of care

Chen et al. (2010) [54]	Retrospective, uncontrolled 25 eyes 3 months	Nepafenac 0.1%	Nepafenac with IVR/IVB for persistent SRF/IRF/PED	VA and CMT unchanged at end of study	No significant change in VA or OCT with the addition of nepafenac

Flaxel et al. (2012) [57]	Randomized, prospective, controlled, 30 eyes 12 months	Bromfenac 0.09%	Bromfenac with IVR (C) versus IVR for new/recurrent exudative AMD	No difference for VA and no. of injections, but CMT decreased more in C group (*P* = 0.03)	Combination therapy with bromfenac may be more efficacious than IVR alone

Gomi et al. (2012) [58]	Randomized, prospective, placebo-controlled, 38 eyes 6 months	Bromfenac 0.1%	Bromfenac with IVR (C) versus IVR for exudative AMD	Fewer injections in C group (*P* = 0.03) VA similar (*P* = 0.31) CMT tended to be lower in C group (*P* = 0.06)	Bromfenac may reduce the need for intravitreal injections

NSAID: nonsteroidal anti-inflammatory drug; AMD: age-related macular degeneration; C: combination; PDT: photodynamic therapy; CNV: choroidal neovascularization; VA: visual acuity; GLD: greatest linear dimension; CMT: central macular thickness; IVR: intravitreal ranibizumab; IVB: intravitreal bevacizumab; SRF: subretinal fluid; IRF: intraretinal fluid; PED: pigment epithelial detachment; OCT: optical coherence tomography.

**Table 2:** Studies treating diabetic macular edema with intravitreal NSAIDs.

Study	Sample size and duration	NSAID	Treatment group(s)	Visual outcomes	Anatomic outcomes
Soheilian et al. (2010) [83]	5 eyes 8 weeks	Diclofenac 500 mcg in 0.1 mL	Diclofenac only (no comparison)	VA improved in 2, worsened in 2, unchanged in 1	CMT worsened in 4 of 5 at 2 weeks, mean CMT worsened at 8 weeks

Reis Ado et al. (2010) [85]	40 eyes 1 month	Ketorolac 500 mcg in 0.1 mL	Ketorolac (20 eyes) versus control (20 fellow eyes)	VA improvement seen in treated eye over fellow eye (*P* = 0.039)	No difference in foveal thickness or macular volume seen between groups

Maldonado et al. (2011) [86]	25 eyes 30 days	Ketorolac 3000 mcg in 0.1 mL	Ketorolac only (no comparison)	VA improved ≥5 letters in 28% at 30 days	No significant improvement in macular thickness

Elbendary and Shahin (2011) [84]	32 eyes 12 weeks	Diclofenac 500 mcg in 0.1 mL	Diclofenac (16 eyes) versus 4 mg IVT (16 eyes)	No difference in final mean VA or improvement Only significant improvement in IVT group	Decreased CMT seen in both groups but not significantly different

NSAID: nonsteroidal anti-inflammatory drug; VA: visual acuity; CMT: central macular thickness; IVT: intravitreal triamcinolone.
